# Hormonal priming, induction of ovulation and in-vitro fertilization of the endangered Wyoming toad (Bufo baxteri)

**DOI:** 10.1186/1477-7827-4-34

**Published:** 2006-06-22

**Authors:** Robert K Browne, Jessica Seratt, Carrie Vance, Andrew Kouba

**Affiliations:** 1 Memphis Zoo, 2000 Prentiss Place, Memphis, TN 38112, USA; 2 Department of Biology, University of Memphis, Memphis, TN 38152, USA

## Abstract

The endangered Wyoming toad (Bufo baxteri) is the subject of an extensive captive breeding and reintroduction program. Wyoming toads in captivity rarely ovulate spontaneously and hormonal induction is used to ovulate females or to stimulate spermiation in males. With hormonal induction, ovulation is unreliable and egg numbers are low. The sequential administration of anovulatory doses of hormones (priming) has increased egg numbers and quality in both anurans and fish. Consequently, we tested the efficacy of a combination of human Chorionic Gonadotrophin (hCG) and Luteinizing Hormone Releasing Hormone analogue (LHRHa) administered as one dose, or two or three sequential doses to Bufo baxteri on egg numbers, fertilization and early embryo development. Spawning toads deposited eggs into Simplified Amphibian Ringers (SAR) solution to enable controlled in-vitro fertilization (IVF) with sperm from hormonally induced male toads. Unprimed females receiving a single mixed normally ovulatory dose of 500 IU hCG plus 4 micrograms of LHRHa produced no eggs. Whereas females primed with this dose and an anovulatory dose (100 IU hCG and 0.8 micrograms of LHRHa) of the same hormones, or primed only with an anovulatory dose, spawned after then receiving an ovulatory dose. Higher total egg numbers were produced with two primings than with one priming. Moreover, two primings produced significantly more eggs from each individual female than one priming. The cleavage rate of eggs was not found to differ between one or two primings. Nevertheless, embryo development with eggs from two primings gave a significantly greater percentage neurulation and swim-up than those from one priming. Of the male toads receiving a single dose of 300 IU hCG, 80% produced spermic urine with the greatest sperm concentration 7 hours post-administration (PA). However, peak sperm motility (95%) was achieved at 5 hours PA and remained relatively constant until declining 20 hours PA. In conclusion, Bufo baxteri egg numbers and quality benefited from sequential priming with LHRHa and hCG whereas spermic urine for IVF was produced from males with a single dose of hCG. The power of assisted reproduction technology in the conservation of endangered amphibians is shown by the release of nearly 2000 tadpoles produced by IVF during this study.

## Background

Amphibian populations worldwide are experiencing unprecedented declines. In the U.S. alone, 55 of 262 amphibian species, or about 21%, are threatened with extinction [1]. One of these threatened species is the Wyoming toad (*Bufo baxteri*) with the last census revealing less than a hundred animals located in the wild [2]. Dr. George Baxter, from the University of Wyoming Zoology department, first discovered the Wyoming toad in 1946 [3]. Although common from the 1950's to the 1970's, a rapid population decline resulted in *B*. *baxteri *being federally listed as endangered in 1984. Several reasons have been suggested as causing the species' decline including pesticides, predation, disease, and habitat modification [4-7]. A Wyoming toad recovery group was formed in 1987 to coordinate habitat protection, environmental monitoring and research. One of the first objectives of the recovery group was to establish a captive breeding program for the long-term management of *B*. *baxteri *[8]. The Wyoming Game and Fish Department's Sybille Wildlife Research Unit first initiated the captive breeding program in 1993 followed by the U.S. Fish and Wildlife Service's Saratoga National Fish Hatchery in 1998. Over the next decade animals were loaned to several zoological institutions in order to aid the recovery and reintroduction efforts.

The recovery group has led one of the most successful captive breeding and reintroduction programs for any endangered anuran, having released over fifty thousand tadpoles to the wild. Wyoming toads are now breeding in their natural habitat and new reintroduction sites have recently been established. Although these successes provide optimism for this species' recovery, captive breeding programs will continue as an important component of the recovery program. Currently, the captive breeding program is limited by low reproductive output due to poor ovulation rates, low egg numbers and low fertilization rates. Hence, the captive breeding program for *B*. *baxteri *would benefit from reproduction technologies that improve reproductive output including the hormonal induction of spermiation and spawning, followed by *in-vitro *fertilization (IVF). The low reproductive output of *B. baxteri *females in captivity is possibly due to poor egg quality as a result of inadequate egg maturation. To find if infertility was caused by a lack of fertilization by the amplexing male a preliminary experiment was coordinated by Dr. Browne at the Saratoga National Fish Hatchery in Wyoming. Essentially, strings of eggs fertilized by an amplexing male were immediately removed and fertilized *in-vitro *by surplus quantities of hormonally induced sperm and compared to fertilization of eggs that did not receive this additional sperm. No significant difference in the treatments between the cleavage or neurulation rates showed that low neurulation rates were not caused by poor fertilization by amplexing males [Browne and Kouba, unpublished].

Initially during the captive breeding program, toads failed to spawn naturally even after attempts to simulate hibernation in captivity. Also historically, during simulated hibernation many toads succumbed to bacterial and fungal infections [6], which led many institutions to attempt artificial reproduction through the use of hormones. Subsequently, an analog of Luteinizing Hormone Releasing Hormone (LHRHa) which induces spawning in fish [9,10] was administered to *B*. *baxteri*. This hormone, at concentrations of 4 μg/50 g body weight, was found to induce spermiation in some male toads [11,12] and ovulation in some females [12]. To date, no fertile egg masses have been produced without at least one sex having received a hormone treatment [pers. comm. RA Odum]. Recently, male toads over-wintered outside in a natural hibernaculum have produced spermatozoa, as shown by high fertilization rates without hormone therapy, suggesting entrainment of reproduction can be achieved by modification of their environment (pers. comm. Jason Palmer).

Of the many different analogs of LHRH commercially available, only D-Ala6, des-Gly10 ethylamide [9], has been found to be effective in inducing spermiation in male toads and frogs [11,13,14] and ovulation in female frogs [15,16]. Human Chorionic Gonadotropin (hCG) has also been shown to be effective in stimulating ovulation in *Xenopus laevis *[17-19], *B. americanus *[Kouba et al., unpublished] and *B. fowleri *[Browne et al., unpublished] and to a limited extent with *Eleutherodactylus coqui *[15]. Although hCG was first used to induce spermiation in live toads nearly 60 years ago for human pregnancy tests [20], its use today in captive breeding programs has primarily been replaced by LHRHa. To our knowledge, there are no published findings directly comparing the efficacy of these two hormones, hCG and LHRHa, on spermiation in a single species. Recent unpublished work from our laboratory has shown that 300 IU to 500 IU of hCG is more effective at inducing spermiation in B. fowleri than LHRHa at 4 :g/50 g body weight.

The captive breeding results for *B*. *baxteri *at the Saratoga National Fish Hatchery from 2001 to 2005 show that female toads ovulate poorly (~50%) and display low hatch numbers when administered only a single dose of LHRHa and therefore could benefit from hormone priming that induces an artificial ovulatory cycle that mimics their natural cycle. In some cases, females were given a second ovulatory dose of LHRHa 24 hrs later, however, egg numbers and fertilization rates were low. Sequential administration of anovulatory 'priming' doses of hormones before administration of a final ovulatory dose increases both egg numbers and egg quality in fish [21] and in some species ovulation has not occurred at all without priming [22]. Furthermore, pre-incubation and priming with hCG of manually isolated follicles from *X. laevis *shortened oocyte maturation times *in-vitro *[23], suggesting *in-vivo *priming could have a similar affect. Therefore, we proposed that in addition to priming, the administration of a combination of LHRHa and hCG might also increase egg numbers and improve egg quality in *Bufo *species such as *B*. *baxteri*. To our knowledge, no experimental designs have previously addressed the combined use of hCG and LHRHa for the induction of ovulation in anurans, nor has hCG been tested for its ability to induce spermiation in male *B. baxteri*.

In the present study, we test the use of priming with a combination of hCG and LHRHa to improve the hormonal induction of ovulation and the efficacy of hCG in the induction of spermiation in *B. baxteri*. Using the oocytes and sperm obtained from hormonal induction, we then attempted *in-vitro *fertilization to assess whether novel reproduction technologies for managing the long-term genetic diversity of endangered anurans could be accomplished through multiple paternities [24]. Female toads were held in Simplified Amphibian Ringers (SAR) solution to extend the fertility of spawned oocytes, which were then artificially fertilized with hormonally induced spermic urine collected from several males. Eggs were then monitored for fertilization (cleavage), neurulation and swim-up rates.

## Materials and methods

### Animals

Wyoming toads were kept in 45 L (10 gallon) glass aquariums with a 25 cm (1") thick sponge mat and a cork bark slab for shelter or basking. Standard fluorescent lights were provided for the aquarium and set on a timer to simulate natural day light hours, and a water tray (10 cm diameter × 2 cm depth) was placed at one end. Tanks and sponge mats were cleaned daily. Four female or six male toads were housed per aquarium and offered a range of food items including mealworms, wax-worms and crickets. The crickets were dusted with Reptivite^© ^powder to provide a variety of vitamins and minerals.

### Hormonal induction of spermiation

The purpose of this experiment was to: 1) collect spermic urine for IVF; and 2) characterize the spermiation response induced in male *B. baxteri *when administered a single dose of 300 IU hCG. A single dose of 300 IU of hCG is optimum for sperm production in *B. americanus *and *B. fowleri *when compared to lower concentrations [Kouba et al., unpublished]. Variables measured in response to hormonal induction include the number of males producing spermatozoa, percentage sperm motility, forward progressive motility (PM), sperm concentration and the volume of urine. Spermatozoa exhibiting beating flagella were considered motile, even if no forward progression was observed. Progressive motility (PM) was based on a scale of 0 to 5, where 0 = no movement and 5 = rapid forward movement.

To induce spermiation, ten male *B. baxteri *(56.4 ± 0.9 mm, 39.6 ± 2.4 g; means ± SE) were administered a dose of 300 IU of hCG (Sigma, St. Louis, C-1063) in 100 μl of reagent grade water by injection in the intra-peritoneal cavity. The toads were then placed individually in 3.8 L plastic boxes with 1 cm of tap water. The water was changed if soiling was apparent. The male toads were then sampled for spermic urine hourly from 3 to 13 hrs post-administration (PA), the period of spermiation when using hCG with *B. americanus *and *B. fowleri *[Kouba et al., unpublished]. To test the maximum duration of hormonally induced spermiation, *B. baxteri *urine was also sampled from 20 to 24 hrs PA and evaluated for the presence of spermatozoa.

To collect spermic urine, toads were carefully removed from their box and excess water was removed with a tissue. The toads were then held by a thumb and index finger, anterior and across the pelvic girdle, above a 150 mm diameter Petri dish. Gentle massaging then promoted urination, usually within 60 seconds. Urine was removed from the dish and placed in a 1.5 ml Eppendorf tube. The volume expressed by each toad at each sampling period was then measured. A 10 μl aliquot was then placed on a slide that had been smeared with 0.5 % (w/v) Bovine Serum Albumin (Sigma Aldrich, St. Louis, A-3311) solution in distilled water to prevent the sperm cells from sticking to the slide [24] and the percent motility and progressive motility (PM) of sperm were measured. The percentage of motile sperm was measured under 1000× magnification and the PM was categorized under 400 × magnification. Only samples showing good motility (>70 %, 3.0 PM) and concentration (>10^6 ^ml^-1^) were used to fertilize eggs. Depending on its availability, spermic urine was used fresh or stored for a short time in the refrigerator on ice (0°C) [25]. The motility of refrigerated sperm was then re-assessed prior to its use in fertilization as previously described. The sperm concentration of each sample was measured using a Neubaeur hemocytometer (to the nearest 0.1 × 10^6 ^ml^-1^).

### Hormonal induction of egg production and the *in-vitro *fertilization of eggs

The purpose of this experiment was to determine if *B. baxteri *ovulate in response to a single mixed dose of 500 IU hCG and 4 μg LHRHa, or if *B*. *baxteri *require a series of doses with these hormones for the final maturation of oocytes. The measured variables were the number of ovulating females, the number of eggs ovulated, and after *in-vitro *fertilization the percent cleavage, neurulation and swim-up. Swim-up is when aquatic amphibian larvae exhaust their yolk sac and 'swim-up' to begin endogenous feeding from their supine position.

Twenty female *B. baxteri *were randomly divided into two groups of ten. One group was administered a single normally 'ovulatory' dose of 500 IU of hCG and 4 μg LHRHa (Sigma Aldrich, St. Louis, L4513). None of these females was induced to spawn and this group's response was considered the unprimed control. Thereafter, this group was considered as having one priming of 500 IU of hCG and 4 μg LHRHa combined. Seventy-two hrs later, both this previously primed group and the group to receive one priming, were administered an 'anovulatory' dose of 100 IU hCG and 0.8 μg LHRHa combined. This dosage is 20% the ovulatory dose and did not induce ovulation in either group previously having received one or no priming. After an additional period of 96 hrs, both groups were administered an normally ovulatory dose of 500 IU of hCG plus 4 μg LHRHa combined, after which ovulation occurred. Consequently, prior to administration of the final ovulatory hormone dose one group of females had received two primings while the other group had received one priming as shown in Table 1. All hormones were administered in 200 μl of sterile water through intra-peritoneal injection. Individual animals were placed into single 11.4 L plastic boxes containing 1.5 cm of Simplified Amphibian Ringers solution (SAR; 6.6 g•l^-1 ^NaCl, 0.147 g•l^-1 ^CaCl_2_, 0.149 g•l^-1 ^KCl, 0.302 g•l^-1 ^NaHCO_3_; 220 mOsmol•kg^-1^; [27] to lengthen the time the eggs would remain fertilizable. The SAR was changed if soiling was apparent and the spawning of eggs monitored. Eleven and half hrs after the ovulatory dose, the first females were observed producing eggs.

**Table 1:** The protocol for hormone administration of none, one or two primings for female *Bufo baxteri *and the hormone type and concentration. No females ovulated when administered a single normally ovulatory hormone dose (500 IU hCG plus 4 micrograms of LHRHa) in the absence of priming (No priming). Nor did females ovulate when administered only an anovulatory hormone dose (100 IU hCG and 0.8 micrograms of LHRHa; One priming), or the normally inducing ovulatory dose and then after 72 hrs the anovulatory dose (Two primings). Females receiving both one and two primings were given thier anovulatory dose 96 hrs before the final ovulating dose resulting in a total time of 168 hrs for females receiving two primings.

		Hormone concentration		
Treatment	Priming dose	Priming dose	Ovulatory dose	Result

Time (hours)	0 hrs	72 hrs	168 hrs	
No priming	500 IU hCG + 4 μg LHRHa			No ovulation
One priming		100 IU hCG + 0.8 μg LHRHa	500 IU hCG + 4 μg LHRHa	Ovulation
Two primings	500 IU hCG + 4 μg LHRHa	100 IU hCG + 0.8 μg LHRHa	500 IU hCG + 4 μg LHRHa	Ovulation

The first eggs were produced between 11.5 to 12.5 hrs PA and were collected at hourly sampling times until 24 hrs PA. At each sampling time, a sub-sample of 100 to 150 eggs from each female were placed in one or more dry Petri dishes. One Petri dish per male was used when fertilization by multiple males was attempted. Approximately 100 μl of urine containing sperm samples of good motility and concentration were pipetted onto the egg string, the eggs and sperm mixed and then left for 10 minutes before flooding the dishes with water.

Fertilization rates were assessed as the percentage of cleaved eggs at larval stage 3 to 6 [28] from 6 to 8 hrs after the application of sperm to the eggs. Counts of cleaved eggs and unfertilized eggs were taken using a stereo dissecting microscope (Omano™) and the percent cleavage was calculated. Two indices of egg/embryo quality were used: 1) the percentage of eggs reaching neurulation, larval stage 26–28 [28]; and 2) the percentage of embryos reaching 'swim-up' (endogenous feeding, larval stage 44–46) [28]. After conclusion of the experiments, tadpoles were released to the U.S. Fish and Wildlife Service as part of the *B. baxteri *reintroduction program.

### Statistics

If more than one male was used to fertilize oocytes at each sampling period, the total number of oocytes in all the dishes from one female was considered the female replicate. Data was compared between treatments as the mean of toad replicates and as a mean of sampling time replicates. Egg numbers, cleavage, neurulation and swim-up rates are expressed as the means ± SE and differences were considered significant at P < 0.05. All percentage data were arcsine transformed prior to analyses and tested for normality and homogeneity of variance using the Shapiro-Wilk W test. If data was normal, Pearson product-moment correlations were tested; Tukey-Kramer least significant difference was used for multiple comparisons of means and Student *t*-tests for pairs of means. If data was non-parametric, a Wilcoxon (Chi-squared) test was used for mean comparisons. All statistical analyses were performed using the JMP 5.1 software package [29].

## Results

### Hormonal induction of spermiation

**Figure 1:**
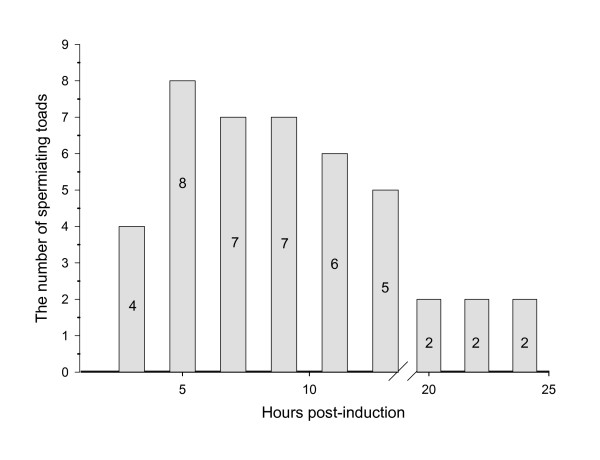
The number of toads (n = 10) spermiating over 25 hrs post-induction to a single intraperitoneal dose of 300IU of hCG.

**Figure 2:**
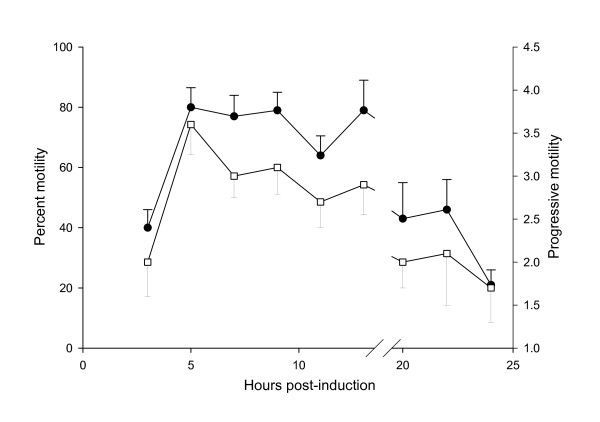
The percentage of motile sperm ●, and their progressive motility □, over the period of spermiation from three to twenty four hrs post-induction. Data shown are means ± SE.

**Figure 3:**
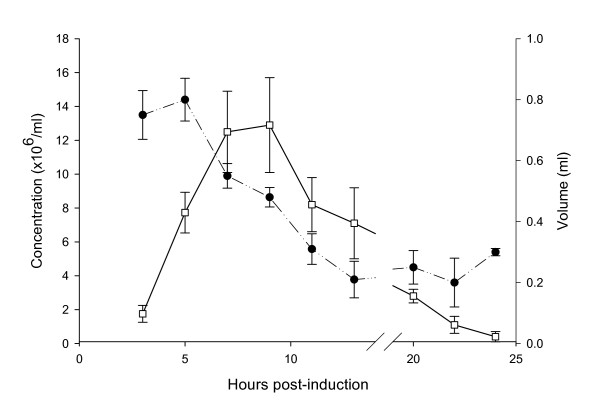
The volume of urine (ml) expressed ●, and the concentration of sperm (×106 ml-1), over the period of spermiation from three to twenty four hrs post-induction. Data shown are means ± SE.

Of the male toads administered hCG 80% (8/10) were induced to produce spermatozoa within 5 hrs of hormone treatment (Figure 1). Although four males were producing sperm 3 hrs PA, we found that sperm motility and PM (22% and 1.4, respectively) was low and another 2 hrs was necessary to achieve maximum motility (95%) and PM (3.4) (Figure 2). Once maximum motility was reached at 5 hrs PA, it remained constant with a drop in motility first observed at 20 hrs PA. Unlike motility, the concentration of sperm peaked at 7 hrs PA and began to decline by 12 hrs PA (Figure 3). Interestingly, the volume of urine that could be obtained from the males declined steadily from 5 hrs PA until 14 hrs PA after which the volume remained constant (Figure 3). Only two males were still producing spermatozoa 20 hrs PA and by 24 hrs PA sperm motility, PM and concentration were very low.

### Hormonal induction of spawning

Females receiving an initial combined ovulatory dose of hCG (500 IU) plus LHRHa (4 μg) without hormone priming failed to spawn any eggs. However, females given either one or two priming hormones were induced to spawn eggs beginning at 11.5 to 12.5 hrs PA of an ovulatory dose. Females that received priming hormones were observed spawning up to 23 hrs PA, stopping in a relatively synchronous manner. After 17.5 hrs PA, both treatment groups continued to spawn eggs; however, none of these eggs were fertilized after being mixed with spermatozoa. Hence, oocytes spawned from 11.5 to 17.5 hrs PA are referred to as falling within the 'fertile period' while oocytes spawned from 20.5 to 23 hrs PA are referred to as falling within the 'non-fertile period'. Oocytes spawned during these two distinct periods were separated into their own groups for analysis and compared. For females receiving one priming, 35% of the total numbers of oocytes spawned were produced during these non-fertile periods (20.5 to 23 hrs PA). In contrast, the number of oocytes produced during the non-fertile period for females receiving two primings was much less at 11% of the total. Of all females only one (from the single primed group) spawned for the first time at 17.5 hrs PA during the non-fertile period.

During the fertile period, females receiving two primings were induced to spawn earlier (11.5 hrs post-induction) when compared to females receiving one priming (12.5 hrs PA) (Table 2). Moreover, the total number of oocytes spawned from 11.5 to 17.5 hrs PA was greater (P < 0.001) in females receiving two primings (22,961) compared to one priming (9,883), and the mean number of oocytes spawned for two primings (3280 ± 159) compared with one priming (1,647 ± 167) was significantly (P > 0.01) different (Table 2). There were also greater total numbers of eggs produced during each individual time point for one priming compared with two primings and significantly more (P < 0.001) toads producing eggs at each sampling time after receiving two primings (6.9 ± 0.1) than with one priming (3.7 ± 0.3). Yet, the mean number of oocytes spawned per individual female was not significant by treatment at any sampling time due to the large variance in oocyte numbers (Table 2). The range of numbers of spawning females during any given time point within the fertile period was less in females having received one priming (0–5) compared to females having received two primings (6–8).

**Table 2:** The number of toads spawning, the mean number of eggs produced per. spawning toad and total eggs spawned during the fertile period (11.5–17.5 hrs post-induction) for *Bufo baxteri *having received one or two primings. Data are expressed as the mean ± SE. There was a significant difference between one or two primings in the mean total number of eggs produced.

	No. toads spawning and egg production with one priming.			No. toads spawning and egg production with two primings.		
Hrs post-administration	No. Toads spawning	No. Eggs (Mean ± SD)	Total Eggs Spawned	No. Toads Spawning	No. Eggs (Mean ± SD)	Total eggs spawned

11.5	0	0	0	8	443 ± 184	3,551
12.5	3	376 ± 176	1,127	7	520 ± 152	3,640
13.5	3	743 ± 242	2,230	7	525 ± 68	3,675
14.5	3	193 ± 17	1,805	7	415 ± 44	2,905
15.5	4	468 ± 175	1,872	7	436 ± 36	3,055
16.5	5	314 ± 70	1,579	6	588 ± 156	3,530
17.5	4	318 ± 94	1,270	7	372 ± 89	2,605
Total No. Eggs			9,883			22,961
Mean ± SE			1,647 ± 167			3,280 ± 159

Overall, there were a significantly greater number of total eggs induced from females administered two priming hormone doses (25,721) vs. one (13,320) (Table 3). Moreover, the number of females that spawned was greater for females administered two primings (88%; 8/9) compared to one priming (70%; 7/10), although this number was not significant. During the fertile period, significantly (P < 0.01) more eggs were produced from two primings (2,551 ± 500) compared to one priming (865 ± 353) for all females within each treatment group and when looking at spawning females only, significantly (P < 0.05) more eggs were produced from two primings compared to one priming (2,870 ± 436, 1,441 ± 465, respectively) (Table 3). However, there were no significant differences in number of eggs spawned between females receiving either one or two primings during the 'non-fertile' period.

**Table 3:** The number of eggs produced during the 11.5 to 17.5 hrs post-induction period when eggs were fertile, the numbers of eggs from 21.5 to 23 hrs post-induction during which eggs were infertile, and the total number of eggs from both periods (means ± SE). One female expired from the treatment group receiving two primings before egg laying commenced, hence there are nine animals for this group compared to ten for the group receiving one priming.

	No. eggs spawned with one priming hrs post-induction.			No. eggs spawned with two primings hrs post-induction.		
Toad Number	11.5–17.5	20.5–23	11.5–23	11.5–17.5	20.5–23	11.5–23

1	0	0	0	3,540	120	3,660
2	250	250	500	3,530	230	3,760
3	1,430	230	1,660	4,355	1,000	5,355
4	2,325	300	2,625	1,191	750	1,941
5	0	0	0	0	0	0
6	260	300	560	2,050	0	2,050
7	1,250	540	1,790	1,255	260	1,515
8	0	1,710	1,710	4,090	0	4,090
9	3,135	1,340	4,475	2,950	400	3,350
10	0	0	0	NA	NA	NA
Total eggs	8,650	4,670	13,320	22,961	2,760	25,721
Average No. eggs for all toads	865 ± 353 (n = 10)	467 ± 186 (n = 10)	1,332 ± 455 (n = 10)	2,551 ± 500 (n = 9)	306 ± 118 (n = 9)	2,857 ± 539 (n = 9)
Average No. eggs of spawning toads	1,441 ± 465 (n = 6)	667 ± 228 (n = 7)	1,903 ± 512 (n = 7)	2,870 ± 436 (n = 8)	460 ± 139 (n = 6)	3,215 ± 458 (n = 8)

### *In-vitro *fertilization and embryo development

**Figure 4:**
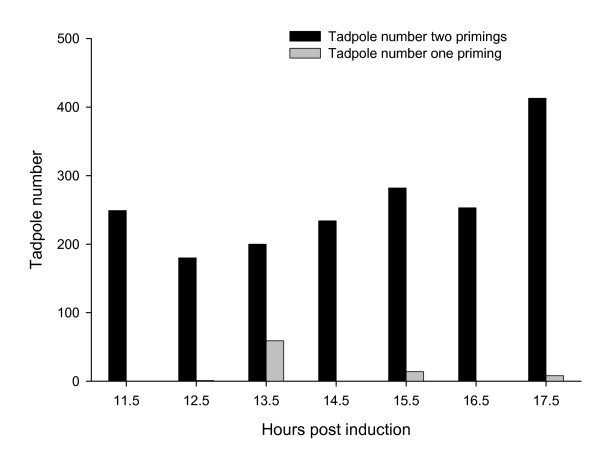
The total number of tadpoles produced with one or two primings at each sampling period. There were significantly (P < 0.05) more tadpoles with two primings at all sampling periods than with one priming.

Oocytes produced during the fertile period of 11.5–17.5 hrs PA were fertilized and evaluated for percent cleavage, neurulation (hatch) and swim-up. The mean number of oocytes available for *in-vitro *fertilization was significantly different (P < 0.01) between females receiving two primings (2,870 ± 436) compared to one priming (1,441 ± 465) (Table 4). Although the percentage of cleaved eggs for the entire fertile period was higher from females receiving two primings compared to one priming (12.7 ± 3.4; 6.4 ± 3.6, respectively) this difference was not significant (Table 4). However, when looking at the mean for the entire fertile period, eggs from females receiving two primings compared to one had a greater (P < 0.01) percent neurulation rate (9.5 ± 2.9 vs. 1.7 ± 0.6) and swim-up rate (6.9 ± 2.3 vs. 0.2 ± 0.1), respectively (Table 4). When percent cleavage, neurulation, and swim-up are evaluated during the individual sampling points (11.5 to17.5 hrs), we found that there was no difference in the percent cleavage for eggs between the priming treatment groups. However, there were differences between periods in percent neurulation and swim-up. Females given two primings produced a greater number of tadpoles (n = 2,300) compared to females given one priming (n = 84). Not surprising, tadpoles were produced during all periods by females administered two primings while those females receiving only one priming were found to produce tadpoles from three periods only, 13.5, 15.5 and 17.5 hrs PA (Figure 4). The lower number of tadpoles observed produced from females administered one priming is the result of lower fertility and viability compared to the other treatment.

**Table 4:** The number of eggs, percent cleavage, neurulation and swim-up during the fertile period (11.5–17.5 hrs post-induction) for *Bufo baxteri *having received either one or two primings. In this table, the mean number of eggs, percent cleavage, neurulation and swim-up are based only on toads that produced eggs. Data are expressed as means ± SE. A significant difference was found for egg numbers, percent neurulation and swim-up, but not for percent cleavage between the two treatment groups.

	Fertilization data from one priming 11.5–17.5 hrs post-induction				Fertilization data from two primings 11.5–17.5 hrs post-induction			
Toad No.	No. Eggs	Cleavage (%)	Neurulation (%)	Swim-up (%)	No. Eggs	Cleavage (%)	Neurulation (%)	Swim-up (%)

1	0	na	na	na	3,540	16.9	13.1	7.2
2	250	0	0	0	3,530	21.7	12.4	11.8
3	1,430	2	1.2	0.6	4,355	24.8	21.1	17.7
4	2,325	3.1	3.1	0	1,191	0	0	0
5	0	na	na	na	0	na	na	na
6	260	9.6	3.1	0	2,050	3.3	0	0
7	1,250	0.4	0.1	0.1	1,255	22.0	19.3	11.6
8	0	na	na	na	4,090	6.7	3.6	1.9
9	3,135	23.3	2.4	0.5	2,950	6.1	6.6	4.8
10	0	na	na	na	-	-	-	-
Mean ± SE	1,441 ± 465	6.4 ± 3.6	1.7 ± 0.6	0.2 ± 0.1	2,870 ± 436	12.7 ± 3.4	9.5 ± 2.9	6.9 ± 2.3

### Release of the *in-vitro *fertilized tadpoles into the wild

Tadpoles generated from these experiments were provided to U.S. Fish and Wildlife service biologists in Cheyenne Wyoming after three weeks of care at the Memphis Zoo and released into the wild as part of the Wyoming toad recovery plan.

## Discussion

Overall, the results of this study showed that in *B. baxteri *spermiation could be successfully induced with hCG and the optimum period of sperm production and sperm quality was between 5 to 12 hrs PA. This sperm could then be used for fertilization directly or when stored refrigerated and unfrozen. Either one or two primings of hCG with LHRHa induced ovulation in females compared to females that recieved a single   ovulatory injection of these two hormones. Two primings produced more oocytes which also   had a greater percentage neurulation and swim-up rate than those from   one priming. The storage of sperm and the novel technique of the spawning of oocytes in SAR enabled the fertilization of oocytes from females with the sperm of many males up to an hour after spawning.

We found that hCG was able to induce spermiation in 80% of the male *B*. *baxteri *that we treated. These results suggest that hCG may be a valid alternative to LHRHa for captive breeding in *Bufo *species that are not responsive to LHRHa. Concentration of sperm peaked at 7 hrs PA with hCG, yet maximal motility was observed at 5 hrs PA. We found sperm concentrations to be nearly seven times higher than those reported for Wyoming toads administered LHRHa [11,31], suggesting hCG is a more effective hormone for inducing spermiation in this species. Recent work in *Eleutherodactylus coqui *found that higher doses of LHRHa (3.2 μg/g of body weight) were required to induce ovulation [15] than typically administered within captive breeding programs of zoos [12]. It is likely that responses to hormones are species-specific and will require testing of various concentrations to optimize their use for individual species in captive breeding programs.

Spermiation when induced by hCG occurred over a limited period of time from 5 to12 hrs PA. Spermiation must be synchronized with the optimum period of ovulation, which occurred from 11.5 to 17.5 hrs PA, otherwise fertility will decrease due to lower sperm concentrations and poor motility. Therefore, when using in-vitro fertilization males should be hormonally induced 6 hrs later than females in order to synchronize gamete release. This window of time may be extended when not using in-vitro fertilization (IVF) since less frequent sperm collections could enable the animals to retain their spermatozoa.

Problems arising from asynchrony of gamete release can also be solved using various reproduction technologies that support IVF. Several technologies and protocols have been developed to assist IVF in *bufonids *including: 1) the short term storage of spermatozoa at cold temperatures [25], 2) the inactivation and reactivation of spermatozoa [14,19,26], 3) cryopreservation [26,32,33], 4) the extended fertility of oocytes at cold temperatures [25], and 5) the extended fertility of oocytes spawned then stored in solutions of high osmolality [19].

The storage of refrigerated unfrozen spermatozoa and the spawning of oocytes into SAR avoided problems with the asynchrony of sperm and oocyte release. *Bufonid *eggs hydrate quickly in water or other solutions with low osmolality, which prevents fertilization [19]. Therefore, the opportunity to conduct IVF is diminished unless solutions with higher osmolality are used [19,25]. Browne et al. [unpublished] showed that *B. fowleri *oocytes spawned into Simplified Amphibian Ringer (SAR) solution (220 mOsm•kg^-1^) remain fertile for up to 12 hrs. Our novel spawning of *B*.*baxteri *oocytes into SAR enabled the use of IVF for up to one hour. The ability of high osmolalities to extend the fertility of oocytes has also been shown with Myobatrachid frogs [34] and *X. laevis *[19]. Hence, the direct spawning of oocytes from other anuran species into SAR solution during IVF protocols may also be used to extend their fertility.

Despite research in our laboratory suggesting that about 60% of *B. fowleri *and *B. americanus *ovulate in response to a single dose of 500 IU hCG, in this study *B. baxteri *failed to respond to a similar treatment. Why the response to hormones was different in *B*. *baxteri *to that typically found in *B*. *fowleri *or *B*. *americanus *is not known. Some insight could have been gained through the assessment of the follicular stage of the ovaries of *B*. *baxteri*. However, ethical limitations on the experimentation on endangered species prevented the assessment of the condition of the ovaries of *B*. *baxteri *in our study. A single normally ovulatory dose of hCG plus LHRHa was not sufficient to induce ovulation, thus it was not surprising that a subsequent single anovulatory dose of the two hormones did not elicit a response. Clearly, because of the success of the final ovulatory dose, the anovulatory dose was low enough to stimulate oocyte maturation but not induce ovulation in either treatment group. We did not have enough females to create additional treatments that test two sequential ovulatory induction doses or two anovulatory induction doses, however this trial should be conducted in the future. Yet, with *B*. *baxteri *our experiments clearly show the importance of priming on oocyte maturation and ovulation in the absence of a natural hibernation period.

In some anurans, a period of low temperature (hibernation) can be essential for ovulation [35,36] as the function of the toad ovary switches from vitellogenic growth to follicular maturation [37]. Possibly, the females' lack of response to a single ovulatory dose of hormone is from the need for a longer hibernation period to finally mature their oocytes and hibernation may have a greater function on oocyte maturation in the *B. baxteri *compared to more temperate climate *bufonids*. Therefore, the use of primings appears to circumvent the natural breeding cycle of this seasonally breeding toad.

To our knowledge, we are the first to publish the use of a combination of two hormones, LHRHa and hCG, for the priming and subsequent ovulation of anurans. Our results showed that two primings produced a greater number of females spawning, oocyte numbers, percent neurulation and swim-up than one priming, although it is uncertain as to why two primings was more effective. Administration of LHRHa stimulates the pituitary to produce the gonadotrophic hormones luteinizing hormone (LH) and follicle-stimulating hormone (FSH) and FSH stimulates progesterone production in amphibian vitellogenic oocytes thus completing final oocyte maturation [38-42]. As suggested by Schuetz [43], the combination of two hormones may provide a more favorable response due to progesterone having a greater effect on oocyte maturation than hCG, while the opposite is true for ovulation. It may be that two primings, given over a longer period of time, recruited a larger number of mature oocytes of better quality while one priming recruited a smaller number of mature oocytes. Browne et al. [44] showed that progesterone influences oocyte maturation when given *in-vivo *but does not stimulate ovulation in *B. fowleri*. Interestingly, in *X*. *laevis *hCG will only act on ooctyes above a certain diameter when promoted by progesterone [45]. Human chorionic gonadotrophin mimics FSH. Therefore, females with suitable numbers of second growth phase oocytes once administered the ovulatory hormones LHRHa or hCG are usually compelled to spawn even without stimulation by males [41,43]. Although hCG was administered in combination with LHRHa during all the primings, LHRHa alone as a priming hormone may have adequately matured oocytes. The hormone hCG could then have been administered as the preferred ovulatory hormone.

In this study, a period of 96 hrs was sufficient to recruit a mature pool of oocytes for ovulation. However, an extended period of priming (two sequential administrations) produced a greater number of eggs of better quality. It is uncertain why this extended time was needed in *B. baxteri*. *Xenopus laevis *can be induced to ovulate 12 to 24 hrs after receiving a priming hormone [19], although it should be noted that the aquatic *Xenopus spp*. are very different from *bufonids*. There are also species-specific differences observed in fish for optimum oocyte maturation time. In carp (*Cyprinus carpio*), 12 hrs of priming is sufficient to induce a normal spawning response [46] while anovulatory doses of LHRHa through slow release implants over weeks to months was found to be optimum in trout [21].

We observed two distinct periods of spawning from both treatment groups, referred to as the 'fertile' and 'non-fertile' period. Any eggs spawned after 20.5 hrs PA were not fertilized and this lack of fertilization was not due to poor sperm quality. The reason for the production of eggs over two distinct periods, and the poor egg quality in the second ovulatory period is unclear. In many pond-breeding anurans there is a natural diurnal rhythm in egg production where inhibition of egg production occurs at dawn [37]. This was the time that egg production ceased before resuming several hrs later. This delay in spawning could have resulted in the infertile eggs.

There are many reviews describing the importance of reproduction technologies, for managing the long-term genetics of mammalian populations held in captivity [47-50]. However, few captive breeding programs have been able to implement such technologies due to challenges associated with low fecundity, internal fertilization and embryo development followed by placental-based fetal gestation. Most amphibians do not have such limitations; hence captive breeding programs can easily incorporate assisted reproduction, including IVF, for managing amphibian genetics. In our study, we were able to fertilize a single female's clutch (1,000s of eggs) with sperm from five different males (this was repeated with eggs collected from several females), clearly showing the potential for rapidly diversifying the genetics of the population. Moreover, at the end of our trials in July 2004, nearly 2,000 endangered tadpoles were released into the wild by USFWS biologists highlighting the application of such technology for reintroduction efforts. The release of this many endangered amphibians or any other animals, produced by IVF, may be a first for any threatened species.

## Conclusion

Currently, technologies are available to incorporate IVF into captive breeding programs for endangered *bufonid *species and may assist in their long-term genetic management. This study showed that extended 'priming' of *B. baxteri *resulted in higher fecundity. Higher fecundity from two primings, when compared to no priming or one priming, occurred through two responses: 1) an increased number of eggs per. toad, and 2) a greater survival of fertilized eggs to swim-up stage. The number of eggs from females receiving two primings was similar to the number of eggs from *B. baxteri *in nature indicating that egg production is not dependent on hibernation. However, considering the low fertilization rates in our study, we cannot make a similar statement for egg quality. It may be that hibernation plays an important role in the *B*. *baxteri *for final oocyte development and our current protocols rushed the recruitment of a large cohort of eggs. Therefore, the development of methods to increase fertilization and embryo survival rates by producing a greater number of mature follicles before ovulation should be prioritized in the development of reproduction technologies for *B. baxteri*.

## References

[B1] Young BE, Stuart SN, Chanson JS, Cox NA, Boucher TM (2004). Disappearing Jewels: The Status of New World Amphibians. NatureServe, Arlington, Virginia.

[B2] U.S. Fish and Wildlife Service (2005). Status of the Wyoming toad. Annual Report.

[B3] Baxter GT (1946). A study of the amphibians and reptiles of Wyoming (Laramie, Wyoming: University of Wyoming Master's thesis).

[B4] Baxter GT, Stromberg MR, Dood SK (1982). The status of the Wyoming toad (*Bufo hemiophrys baxteri*). Environ Conserv.

[B5] Lewis DL, Baxter GT, Johnson KM, Stone MD (1985). Possible extinction of the Wyoming toad, *Bufo hemiophrys baxteri*. J Herpetol.

[B6] Taylor SK, Williams ES, Thorne ET, Mills KW, Withers DI, Pier AC (1999). Causes of mortality of the Wyoming toad. Wildlife Disease.

[B7] Jennings M, Beiswinger R, Corn S, Parker M, Pessier A, Spencer B, Miller PS, Conservation Breeding Specialist Group (CBSG) of the IUCN (2001). Population and Habitat Viability Assessment for the Wyoming Toad (*Bufo baxteri*). Final Workshop Report.

[B8] Stone MD, U.S. Fish and Wildlife Service (1991). Wyoming toad recovery plan. US Fish and Wildlife Service, Denver, Colorado.

[B9] Arimura A, Vilchez-Martinez JA, Coy DH, Coy EJ, Hirotsu Y, Schally AV (1974). [D-Ala6, Des-Gly-NH210]-LH-RH-ethylamide: a new analogue with unusually high LH-RH/FSH-RH activity. Endocrinol.

[B10] Lam TJ, Pandey S, Hoar WS (1975). Induction of ovulation in goldfish by synthetic luteinizing hormone-releasing hormone(LH-RH). Can J Zool.

[B11] Obringer AR, Brien JK, Saunders RL, Yamamoto K, Kikuyama S, Roth TL (2000). Characterization of the spermiation response, luteinizing hormone release and sperm quality in the American toad (*Bufo americanus*) and the endangered Wyoming toad (*Bufo baxteri*). Reprod Fertil Develop.

[B12] Lipps G, Odum RA The struggle to save the Wyoming toad *Bufo baxteri *: a case history in captive propagation and conservation. Abstracts from the 25th anniversary meeting of the International Herpetological Symposium 2001, Detroit, Michigan.

[B13] Waggener WL, Carroll EJ (1998). A Method for hormonal induction of sperm release in anurans (eight species) and In vitro fertilization in *Lepidobatrachus *species. Develop Growth Differ.

[B14] Kouba AJ, Vance CK, Frommeyer MA, Roth TL (2003). Structural and functional aspects of *Bufo americanus *spermatozoa: effects of inactivation and reactivation. J Exp Zool.

[B15] Michael SF, Buckley C, Toro E, Estrada AR, Vincent S (2004). Induced ovulation and egg deposition in the direct developing anuran *Eleutherodactylus coqui*. Reprod Biol and Endocrinol.

[B16] Toro E, Michael SF (2004). In vitro fertilization and artificial activation of eggs of the direct-developing anuran *Eleutherodactylus coqui*. Reprod Biol and Endocrinol.

[B17] Bellerby CW (1934). A rapid test for the diagnosis of pregnancy. Nature.

[B18] Shapiro HA, Zwarenstein H (1934). A rapid test for pregnancy on *Xenopus laevis*. Nature.

[B19] Hollinger TG, Corton GL (1980). Artificial fertilization of gametes from the South African clawed frog (*Xenopus laevis*). Gamete Res.

[B20] Galli Mainini (1947). Ovulacion del *Bufo arenarum *con gonadotrofina corionica. Revista de la Sociedad Argentina de Biologica.

[B21] Crim LW, Sherwood NM, Wilson CE (1988). Sustained hormone release: II. Effectiveness of LHRH analogue (LHRHa) administration by either single time injection or cholesterol pellet implantation on plasma gonadotrophin levels in a bioassy model fish, the juvenile rainbow trout. Aquaculture.

[B22] Bailey R, Cole B (1999). Spawning the Tinfoil Barb *Barbodes schwanenfeldi *in Hawaii. Center for tropical and sub-tropical Aquaculture.

[B23] LaMarca MJ, Westphal LM, Rein DA (1985). Gonadotropins and the timing of progesterone-induced meiotic maturation of *Xenopus laevis *oocytes. Develop Biol.

[B24] Holt WV, Watson PF, Holt WV (2001). Genetic resource banking and maintaining biodiversity. Cryobanking the Genetic Resource: Wildlife conservation for the future?.

[B25] Browne RK, Clulow J, Mahony M (2001). Short-term storage of cane toad (*Bufo marinus*) gametes. Reproduction.

[B26] Browne RK, Clulow J, Mahony M, Clark A (1998). Successful recovery of motility and fertility of cryopreserved cane toad (*Bufo marinus*) sperm. Cryobiology.

[B27] Rugh R (1962). Culturing of amphibian embryos. 'Experimental Embryology: Techniques and Procedures.

[B28] Nieuwkoop PD, Faber J, eds (1994). Normal Table of *Xenopus Laevis *(Daudin, 1956): A Systematical and Chronological Survey of the Development from the Fertilized Egg Till the End of Metamorphosis.

[B29] SAS Institute (2003). JMP Inc. Version 5.

[B30] Galli Mainini (1947). Ovulacion del *Bufo arenarum *con gonadotrofina corionica. Revista de la Sociedad Argentina de Biologica.

[B31] Roth TL, Obringer AR, Holt WV, Pickard AR, Rodger JC, Wildt DE (2003). Reproductive research and the worldwide amphibian extinction crisis. Reproductive Science and Integrated Conservation.

[B32] Browne RK, Davis J, Pomering M, Clulow J (2002). Storage of cane toad (*Bufo marinus*) sperm for 6 days at 0°C with subsequent cryopreservation. Reprod Fertil and Develop.

[B33] Browne RK, Clulow J, Manony M (2002). The short-term storage and cryopreservation of spermatozoa from hylid and myobatrachid frogs. Cryo Letters.

[B34] Edwards DL, Mahony MJ, Clulow J (2004). Effect of sperm concentration, medium osmolality and oocyte storage on artificial fertilization success in myobatrachid frog (*Limnodynastes tasmaniensis*). Reprod Fertil and Develop.

[B35] Kim JW, Im WB, Choi HH, Ishii S, Kwon HB (1998). Seasonal fluctuations in pituitary gland and plasma levels of gonadotropic hormones in Rana. Gen Comp Endocrinol.

[B36] Tchou SU, Wang YL (1963). The oogenesis sequence and the impossibility of ovular maturation in the female toad raised in an environment having a high temperature for an entire year. Sci Sin.

[B37] Jørgensen CB, Feder ME, Burggen WW (1992). Growth and Reproduction. Environmental Physiology of the Amphibians.

[B38] LaMarca MJ, Westphal LM, Rein DA (1985). Gonadotrophins and the timing of Progesterone-induced meiotic maturation of *Xenopus laevis *eggs. Develop Biol.

[B39] De Albuja CM, Campos M, Del Pino EM (1983). Role of progesterone on eggs maturation in the egg-brooding hylid frog *Gastrotheca riobambea *(Fowler). J Exp Zool.

[B40] Fortune JE (1983). Steroid production by *Xenopus *ovarian follicles at different developmental stages. Develop Biol.

[B41] Wright PA (1961). Induction of ovulation In vitro in *Rana pipiens *with steroids. Gen and Comp Endocrinol.

[B42] Wright PA (1971). 3-keto-delta-4 steroid: requirement for ovulation in Rana pipiens. Gen Comp Endocrinol.

[B43] Schuetz AW (1971). In vitro induction of ovulation and oocyte maturation in *Rana pipien *ovarian follicles: effects of steroidal and nonsteroidal hormones. J Exp Zool.

[B44] Browne RK, Hong L, Seratt J, Kouba A (2006). Progesterone improves the number and quality of hormone induced Fowler toad (*Bufo fowleri*) eggs. Reprod Biol and Endocrinol.

[B45] Reynhout JK, Taddei C, Smith LD, LaMarca MJ (1975). Response of large eggs of *Xenopus laevis *to progesterone In vitro in relation to eggs size and time after previous hCG-induced ovulation. Develop Biol.

[B46] Kumarasiri WS, Seneviratne P (1988). Induced multiple spawning of Chinese Carps in Sri Lanka. Aquaculture.

[B47] Wildt DE, Monfort SL, Donoghue AM, Johnston LA, Howard J (1992). Embryogenesis in conservation biology – or, how to make an endangered species embryo. Theriogenology.

[B48] Lasley BL, Loskutoff NM, Anderson GB (1994). The limitation of conventional breeding programs and the need and promise of assisted reproduction in nondomestic species. Theriogenology.

[B49] Loskutoff NM, Bartels P, Meintjes M, Godke RA, Schiewe MC (1995). Assisted reproduction technology in nondomestic ungulates: a model approach to preserving and managing genetic diversity. Theriogenology.

[B50] Bainbridge DRJ, Jabbour HN (1998). Potential of assisted breeding techniques for the conservation of endangered mammalian species in captivity: a review. Vet Rec.

